# Temporal Dynamic of the Ratio between Monopartite Begomoviruses and Their Associated Betasatellites in Plants, and Its Modulation by the Viral Gene *βC1*

**DOI:** 10.3390/v15040954

**Published:** 2023-04-13

**Authors:** Yi-Jie Wu, Yi-Ming Liu, Heng-Yu Li, Shu-Sheng Liu, Li-Long Pan

**Affiliations:** 1 Ministry of Agriculture Key Lab of Molecular Biology of Crop Pathogens and Insects, Key Laboratory of Biology of Crop Pathogens and Insects of Zhejiang Province, Institute of Insect Sciences, Zhejiang University, Hangzhou 310058, China; wuyijie501@163.com (Y.-J.W.); liufangfang1@163.com (Y.-M.L.); hengyuli@zju.edu.cn (H.-Y.L.); shshliu@zju.edu.cn (S.-S.L.); 2 The Rural Development Academy, Zhejiang University, Hangzhou 310058, China

**Keywords:** monopartite begomovirus, betasatellite, quantitative relationship, *βC1*, tobacco curly shoot virus, cotton leaf curl multan virus

## Abstract

The begomovirus–betasatellite complex constantly threatens crops in Asia. However, the quantitative relationship between begomoviruses and betasatellites remains largely unknown. The quantities of tobacco curly shoot virus (TbCSV) and its betasatellite (TbCSB) and their ratio varied significantly in initial infection, and thereafter, the ratio tended to become constant. The TbCSB/TbCSV ratio in agrobacteria inoculum significantly affected that in plants in the initial infection but not thereafter. Null-mutation of *βC1* that encodes a multifunctional protein important for pathogenesis in TbCSB significantly reduced the TbCSB/TbCSV ratio in plants. Viral inoculum plants with higher TbCSB/TbCSV ratios promoted whitefly transmission of the virus. The expression of *AV1* encoded by TbCSV, *βC1* encoded by TbCSB and the *βC1*/*AV1* ratio varied significantly in the initial infection and thereafter the ratio tended to become constant. Additionally, the temporal dynamics of the ratio between another begomovirus and its betasatellite was similar to that of TbCSV and was positively regulated by *βC1*. These results indicate that the ratio between monopartite begomoviruses and betasatellites tend to become constant as infection progresses, and is modulated by *βC1*, but a higher betasatellite/begomovirus ratio in virally inoculated plants promotes virus transmission by whiteflies. Our findings provide novel insights into the association between begomoviruses and betasatellites.

## 1. Introduction

In recent decades, the production of many crops has been increasingly threatened by plant viral diseases [1]. In tropical, subtropical and warm temperate regions, geminiviruses (family *Geminiviridae*), the largest group of plant viruses, have caused many devastating crop disease epidemics [2,3,4]. Among the 14 genera in the family *Geminiviridae*, the genus *Begomovirus* comprises the largest number of viral species [5,6]. Additionally, members of the genus *Begomovirus* exhibit the widest geographic distribution [2,3,4,7]. Begomoviruses can infect many important dicotyledonous crops such as tomato, cotton and cassava, causing yield losses of up to 100% [2,3,4]. Under field conditions, begomoviruses are essentially transmitted by whiteflies of the *Bemisia tabaci* complex [8,9]. Begomoviruses are classified into monopartite and bipartite according to their number of genomic ssDNA components [5,7]. In Asia, many begomoviruses in the field are constantly associated with subgenomic components, called satellites [3,4,10].

There are three types of satellites associated with begomoviruses, namely alphasatellite, betasatellite and deltasatellite [10,11,12], but see another related reference [13]. Among them, betasatellites have been subjected to extensive investigations due to their well-established role in pathogenesis [10,11,12]. Betasatellites are a group of circular single-stranded DNA molecules with a length of approximately half of that of the associated begomoviruses (around 1.3 kb) [10,14,15]. Sequence analysis of betasatellites revealed the presence of three conserved regions, namely an adenine-rich region, a satellite conserved region (SCR) containing the hairpin loop structure and an open reading frame encoding multifunctional protein βC1 [14,16]. Recently, an additional open reading frame that encodes a novel βV1 protein was found in the viral strand of more than one-third of the reported betasatellites [17].

During the infection of begomoviruses, betasatellites are often required for the suppression of host defense responses and induction of typical symptoms [10,11,12]. Recently, a betasatellite was shown to compromise *Ty-1*-mediated resistance against geminiviruses [18]. βC1 encoded by betasatellites plays a key role in reprograming plant cellular processes and serves as the target and repressor of many kinds of host defense responses such as gene silencing, autophagy and the ubiquitin proteasome system [19]. βV1 protein encoded by betasatellites was found to induce leaf mosaic, chlorosis and hypersensitive response (HR)-type cell death, and contribute to virus infection [17]. Furthermore, betasatellites rely on their helper begomoviruses for replication, encapsidation, whitefly transmission and within-plant movement [12,14]. Therefore, for begomovirus–betasatellite complexes, coordinated action of begomoviruses and betasatellites is required to achieve productive infection and transmission [10,14,15]. However, many aspects of the molecular association between begomoviruses and betasatellites remain largely unexplored. For example, what is the quantitative relationship between begomoviruses and betasatellites during virus infection and transmission, and how and why it is regulated? Investigations of these issues may shed new light on the pathogenesis of the begomovirus–betasatellite complex and provide potential targets for the management of viral diseases.

In this study, we explored the quantitative relationship between begomoviruses and their associated betasatellites during virus infection and transmission. First, we characterized the temporal dynamics of quantities of tobacco curly shoot virus (TbCSV) and its betasatellite (TbCSB) and the TbCSB/TbCSV ratio in natural host common tobacco plants and the model plant *Nicotiana benthamiana* [20,21]. Second, we explored the modulation of quantities of TbCSV and TbCSB and the TbCSB/TbCSV ratio in plants using the TbCSB/TbCSV ratio in agrobacteria inoculum and *βC1* null-mutation. Third, we analyzed the impacts of the TbCSB/TbCSV ratio in the plants as source of inoculum on virus transmission by whiteflies. Fourth, we monitored the expression dynamics of genes encoded by TbCSV and TbCSB, and their ratio in plants. In addition, we investigated the temporal dynamics of the quantities of cotton leaf curl Multan virus (CLCuMuV) and its betasatellite (CLCuMuB) and the CLCuMuB/CLCuMuV ratio, and their responses to *βC1* null-mutation. Our findings provide new insights into the association between begomoviruses and betasatellites, and help to decipher the pathogenesis of the begomovirus–betasatellite complex.

## 2. Material and Methods

### 2.1. Viruses, Plants and Insects

Two viruses, namely TbCSV and CLCuMuV, and their associated betasatellites were analyzed. The GenBank accession numbers are AJ420318 for TbCSV, AJ421484 for TbCSB, JN968573 for CLCuMuV and JN968574 for CLCuMuB. Infectious clones of these begomoviruses and betasatellites were provided by Professor Xueping Zhou, Institute of Biotechnology, Zhejiang University [21,22]. For agroinoculation, agrobacteria (strain EHA105) containing infectious clones were first cultured until OD600 reached 1.9–2.0, and then were resuspended in resuspension buffer containing 10 mM MgCl_2_ (Sangon Biotech, Shanghai, China), 10 mM 2-Morpholinoethanesulfonic acid (Sangon Biotech, China) and 200 μM acetosyringone (Bio Basic Inc., Vancouver, BC, Canada). Unless specified otherwise, equal quantities of agrobacteria containing infectious clones of begomovirus and betasatellite were mixed and used for inoculation. For TbCSV, its natural host plant, common tobacco (*Nicotiana tabacum* cv. NC89), and the model plant *N. benthamiana* were used. For CLCuMuV, only the model plant *N. benthamiana* was tested. These plants were inoculated when they reached 3–4 true-leaf stage, and 1 mL syringes were used to introduce agrobacteria into true leaves. Cotton plants (*Gossypium hirsutum* cv. Zhe-Mian 1793) were used for whitefly rearing. All plants were grown in an insect-proof greenhouse under 26–28 °C, 14:10 light/dark, 60–80% relative humidity. For insects, a colony of Mediterranean (MED) whiteflies (mt*COI* GenBank accession number: GQ371165) of the *B*. *tabaci* complex was used. Only female whiteflies within 0–3 days post-emergence were used for virus acquisition and transmission.

### 2.2. Extraction of Total DNA and RNA and Quantification of Begomoviruses, Betasatellites and Gene Expression Level

Total DNA was extracted with cetyl-trimethyl-ammonium bromide (CTAB) (Sangon Biotech, China). Briefly, leaf samples were first ground and mixed with CTAB solutions containing 2% β-mercaptoethanol (Sigma, Saint Louis, MO, USA). Next, these samples were incubated at 65 °C and then trichloromethane (Sangon Biotech, China) was added. Samples were then centrifuged, upper phase was collected and mixed with isopropanol (Sangon Biotech, China). DNA was obtained by centrifugation and washing with 75% ethanol solution. Total RNA was extracted using TRIzol (Ambion, Austin, TX, USA). Samples were first ground and mixed with TRIzol. Trichloromethane was added and samples were then centrifuged. Supernatants were collected and mixed with isopropanol. After washing with ethanol solution, RNA was obtained by centrifugation. For the quantification of begomoviruses and betasatellites in DNA samples, SYBR Green Premix Pro Taq HS qPCR Kit (Accurate Biology, Changsha, China) and CFX96 Real-Time PCR Detection System (Bio-Rad, Hercules, California, USA) were used. For the analysis of gene expression level of *AV1* and *βC1*, cDNA was synthesized using Evo M-MLV RT Kit with gDNA Clean for qPCR (Accurate Biology, China). QPCR was then performed as mentioned above. Primers are listed in Table 1.

### 2.3. Analysis of the Temporal Dynamics of the Quantities of Begomoviruses and Betasatellites and the Expression of AV1 and βC1 in Plants

Common tobacco and *N. benthamiana* plants were used for TbCSV, and *N. benthamiana* plants were used for CLCuMuV. Plants were first inoculated with a mixture of equal quantities of agrobacteria containing infectious clones of begomoviruses and betasatellites. Next, at 6, 12, 18, 24 and 30 days post-inoculation (dpi), the first fully expanded leaves were sampled and subjected to the analysis of the quantities of begomoviruses and betasatellites and the expression of *AV1* and *βC1*. All plants were sampled only once.

### 2.4. Analysis of the Effects of the TbCSB/TbCSV Ratio in Agrobacteria Inoculum on the Quantities of TbCSV and TbCSB and the TbCSB/TbCSV Ratio in Plants

To prepare agrobacteria inoculum with different TbCSB/TbCSV ratios (1/4, 1 and 4) while keeping constant the final OD600 value of agrobacteria containing infectious clones of TbCSV, agrobacteria containing infectious clones of TbCSV were mixed with different quantities of agrobacteria containing infectious clones of TbCSB. Common tobacco plants were then inoculated and sampled for analysis at 6, 18 and 30 dpi.

### 2.5. Analysis of the Effects of βC1 Null-Mutation on the Quantities of Begomoviruses and Betasatellites and the Betasatellite/Begomovirus Ratio

TbCSB and CLCuMuB were first cloned and ligated into pUC19. Next, mutagenesis that transformed the *βC1* start codon (ATG) to stop codon (TAG) was conducted using Fast Mutagenesis System (Transgen, Beijing, China). Primers are listed in Table 1. Infectious clones of mutant betasatellites were then constructed as described before [21,22]. Equal quantities of agrobacteria containing infectious clones of begomovirus and betasatellite (wild-type or mutant) were mixed and used for inoculation. Agroinoculation was performed as mentioned above. For TbCSV, two experiments were conducted and its natural host plant common tobacco was inoculated. In the first experiment, plants were sampled at 30 dpi and in the second plants were sampled at 18, 30, 42 and 54 dpi. For CLCuMuV, *N. benthamiana* plants were inoculated and the plants were sampled at 18 dpi. Extraction of DNA and quantification of begomoviruses and betasatellites were conducted as mentioned above.

### 2.6. Analysis of the Effects of TbCSB/TbCSV Ratio in the Plants as Source of Inoculum on Whitefly-Mediated Virus Transmission

A total of 2 sets of common tobacco plants were prepared, namely plants that were at 6 and 18 days post-inoculation with the mixture of equal quantities of agrobacteria containing infectious clones of TbCSV and TbCSB. Virus acquisition and transmission were conducted as described before [23]. Briefly, whiteflies were released onto these plants to acquire the virus for two days. Some of the viruliferous whiteflies were collected in groups of 10, and then lysed in lysis buffer (50 mmol/L KCl, 10 mmol/L Tris, 0.45% Tween 20, 0.2% gelatin, 0.45% Nonidet P-40, 60 mg/L proteinase K with pH at 8.4). Quantification of TbCSV and TbCSB was then performed as mentioned above. The remaining whiteflies were transferred to common tobacco seedlings to transmit the virus for two days. Whiteflies were enclosed in leaf-clip cages [24] for virus transmission and five whiteflies were used per plant. After virus transmission, whiteflies were removed and imidacloprid (20 mg/L) was sprayed. At 18 and 30 days after whitefly-mediated virus inoculation, plants were sampled and subjected to the quantification of TbCSV and TbCSB. Additionally, at 18 and 30 days after whitefly-mediated inoculation, test plants were examined for virus-infection symptom and for the presence of viral DNA using PCR.

### 2.7. Statistical Analysis

For the analysis of quantities of begomoviruses and betasatellites, and gene expression level, real time data were calculated using 2^−△Ct^ as normalized to that of plant or whitefly *actin*. All percentage data were arcsine square root transformed for comparison and back-transformed for presentation. For statistical analyses, when three or more treatments were conducted, one-way analysis of variance (ANOVA) along with Fisher’s least significant difference (LSD) was used. When only two treatments were conducted, Student’s independent *t*-test was used for comparison. Differences between treatments are considered significant when *p* < 0.05. All data are presented as the mean ± standard error of mean (mean ± SEM). All statistical analyses were conducted using SPSS 20.0 Statistics and EXCEL.

## 3. Results

### 3.1. Symptoms Induced by TbCSV and TbCSB in Common Tobacco Plants

**Figure 1:**
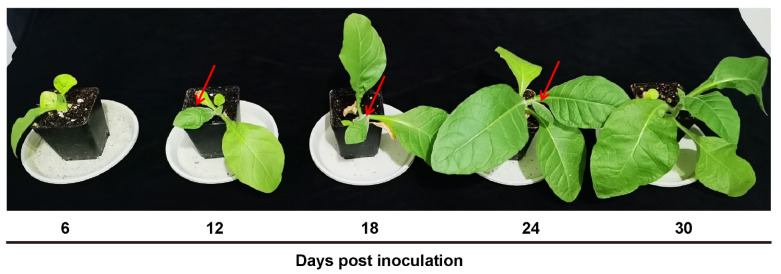
Temporal dynamics of symptoms induced by TbCSV and TbCSB in common tobacco plants. Common tobacco plants were inoculated with a mixture of equal quantities of agrobacteria containing infectious clones of TbCSV and TbCSB, and pictured at designated dpi. Arrows indicate leaves with typical symptoms.

Common tobacco plants, the natural host of TbCSV, were inoculated with a mixture of equal quantities of agrobacteria containing infectious clones of TbCSV and TbCSB. Symptoms (arrowed) induced by TbCSV and TbCSB included downward leaf curling, leaf puckering and vein thickening as previously described [21]. Symptoms thatwere not evident at 6 dpi, became obvious at 12, 18 and 24 dpi, and then became mild at 30 dpi (Figure 1).

### 3.2. Temporal Dynamics of the Quantities of TbCSV and TbCSB and the TbCSB/TbCSV Ratio in Plants

**Figure 2:**
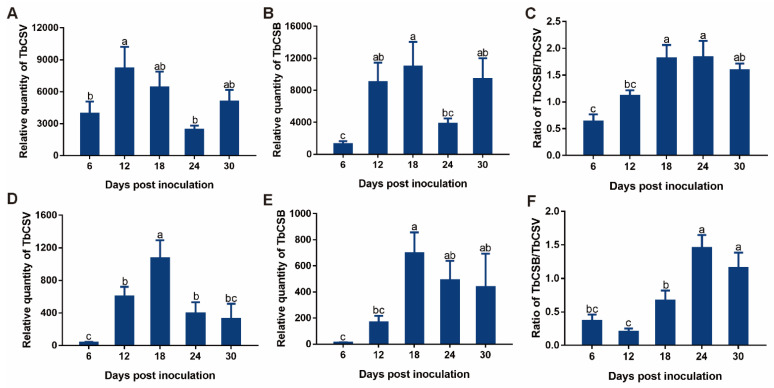
Temporal dynamics of the quantities of TbCSV and TbCSB and the TbCSB/TbCSV ratio in plants. Common tobacco and *N. benthamiana* plants were inoculated with a mixture of equal quantities of agrobacteria containing infectious clones of TbCSV and TbCSB, and then sampled for analysis at designated dpi. Relative quantities of TbCSV and TbCSB and the TbCSB/ TbCSV ratio in common tobacco plants (**A**–**C**) and *N. benthamiana* plants (**D**–**F**). Values are means ± SEM (n = 8–14). Different letters above the columns indicate significant differences (one-way ANOVA, *p* < 0.05).

In natural host, common tobacco plants, TbCSV quantity increased significantly from 6 to 12 dpi, decreased significantly from 12 to 24 dpi and then did not show significant change from 24 to 30 dpi (Figure 2A). TbCSB quantity increased significantly from 6 to 18 dpi, decreased significantly from 18 to 24 dpi and then did not show significant change from 24 to 30 dpi (Figure 2B). The TbCSB/TbCSV ratio increased sequentially from 6 to 18 dpi, and then did not show significant change from 18 to 30 dpi (Figure 2C). When *N. benthamiana* plants were inoculated, TbCSV quantity increased significantly from 6 to 18 dpi, decreased significantly from 18 to 24 dpi and then did not show significant change from 24 to 30 dpi (Figure 2D). TbCSB increased significantly from 6 to 18 dpi, and then did not show significant change from 18 to 30 dpi (Figure 2E). The TbCSB/TbCSV ratio did not show significant change from 6 to 12 dpi, increased sequentially from 12 to 24 dpi and then did not show significant change from 24 to 30 dpi (Figure 2F).

### 3.3. Effects of the TbCSB/TbCSV Ratio in Agrobacteria Inoculum on the Quantities of TbCSV and TbCSB and the TbCSB/TbCSV Ratio in Plants

**Figure 3:**
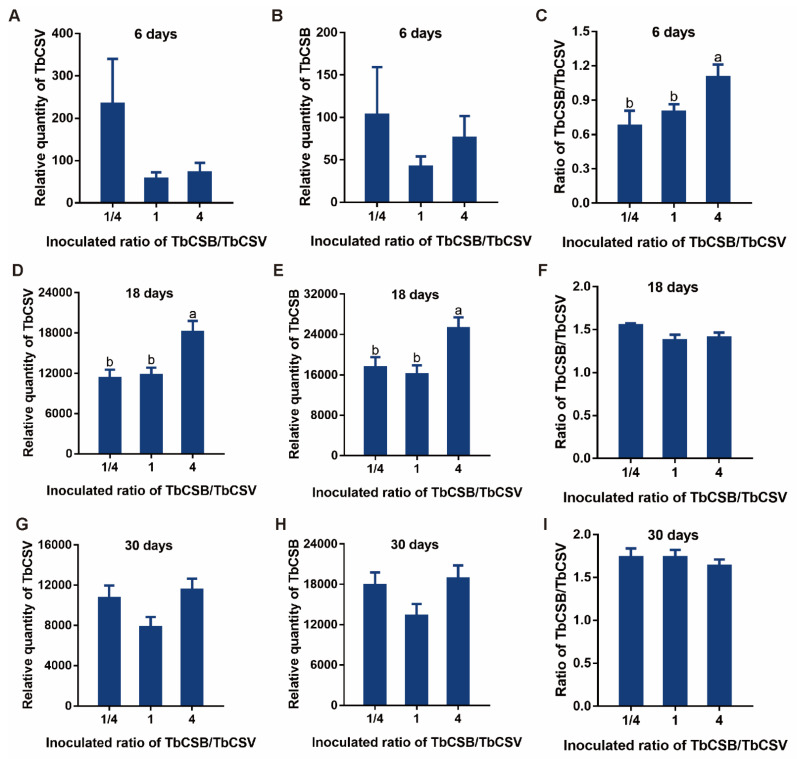
Effects of the TbCSB/TbCSV ratio in agrobacteria inoculum on the quantities of TbCSV and TbCSB satellites and the TbCSB/TbCSV ratio in plants. While keeping constant the final OD600 value of agrobacteria containing infectious clones of TbCSV, agrobacteria containing infectious clones of TbCSV were mixed with different quantities of agrobacteria containing infectious clones of TbCSB to obtain agrobacteria inoculum with different TbCSB/TbCSV ratios (1/4, 1 and 4). Common tobacco plants were then inoculated and sampled for analysis. Relative quantities of TbCSV and TbCSB and the TbCSB/TbCSV ratio at 6 dpi (**A**–**C**), 18 dpi (**D**–**F**) and 30 dpi (**G**–**I**). Values are means ± SEM (n = 11–15). Different letters above the columns indicate significant differences (one-way ANOVA, *p* < 0.05).

Common tobacco plants were inoculated with agrobacteria inoculum with different TbCSB/TbCSV ratios (1/4, 1 and 4), and in these inoculum the final OD600 value of agrobacteria containing infectious clones of TbCSV was kept constant. At 6 dpi, no significant differences in the quantities of TbCSV and TbCSB were found among the 3 treatments (Figure 3A,B). However, the TbCSB/TbCSV ratio was significantly higher in plants inoculated with agrobacteria inoculum with the highest TbCSB/TbCSV ratio (Figure 3C). At 18 dpi, the quantities of TbCSV and TbCSB were significantly higher in plants inoculated with agrobacteria inoculum with the highest TbCSB/TbCSV ratio (Figure 3D,E), but no significant difference in the TbCSB/TbCSV ratio was found among the three treatments (Figure 3F). At 30 dpi, no significant difference in the quantities of TbCSV and TbCSB and the TbCSB/TbCSV ratio was found among the 3 treatments (Figure 3G–I).

### 3.4. Effects of βC1 Null-Mutation on the Quantities of TbCSV and TbCSB and the TbCSB/TbCSV Ratio in Common Tobacco Plants

**Figure 4:**
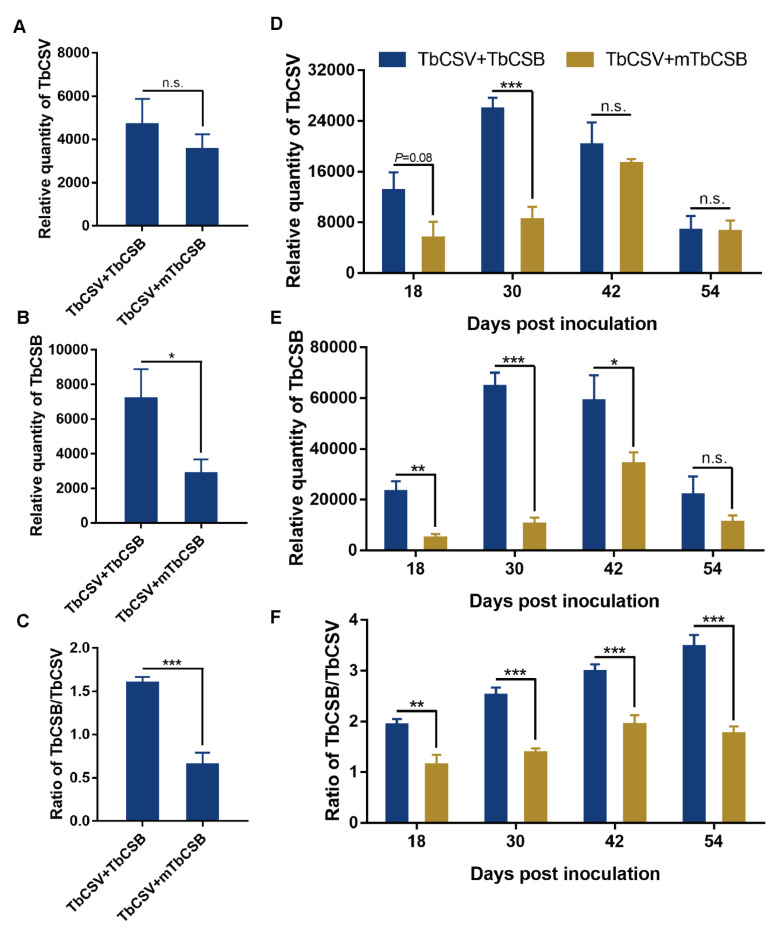
Effects of *βC1* null-mutation on the quantities of TbCSV and TbCSB and the TbCSB/TbCSV ratio in plants. Common tobacco plants were inoculated with a mixture of equal quantities of agrobacteria containing infectious clones of TbCSV and TbCSB, and then sampled for analysis at designated dpi. mTbCSB stands for mutant TbCSB. Two experiments were conducted. Sampling was conducted at 30 dpi in the first experiment (**A**–**C**), and in the second sampling was conducted at a series of time points (**D**–**F**). Values are means ± SEM (n = 9–14). Asterisks indicate significant differences (independent *t*-test, * *p* < 0.05, ** *p* < 0.01, *** *p* < 0.001) and n.s. indicates no significant difference.

Two experiments were conducted to examine the effects of *βC1* null-mutation. In the first experiment, sampling and analysis were conducted at 30 dpi. *βC1* null-mutation did not affect TbCSV quantity, but significantly reduced TbCSB and the TbCSB/TbCSV ratio (Figure 4A–C). We then repeated the experiment and extended our observation to 54 dpi. *βC1* null-mutation did not affect TbCSV quantity at 18, 42 and 54 dpi, but significantly reduced TbCSV quantity at 30 dpi (Figure 4D). *βC1* null-mutation significantly decreased TbCSB quantity at 18, 30 and 42 dpi, but not at 54 dpi (Figure 4E). At all time points analyzed, *βC1* null-mutation significantly reduced TbCSB/TbCSV ratio in common tobacco plants (Figure 4F).

### 3.5. Effects of the TbCSB/TbCSV Ratio in the Plants as Source of Inoculum on Whitefly-Mediated Virus Transmission

**Figure 5:**
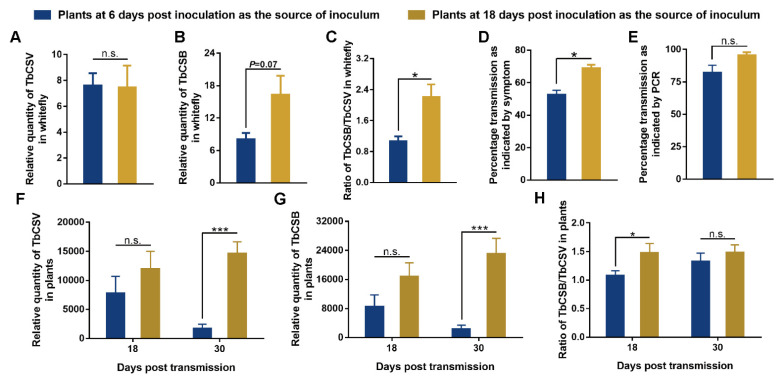
Effects of the TbCSB/TbCSV ratio in the plants as source of inoculum on whitefly-mediated TbCSV transmission. Common tobacco plants that were 6 and 18 days post-inoculation with a mixture of equal quantities of agrobacteria containing infectious clones of TbCSV and TbCSB were presented to whiteflies for oral virus acquisition. Viruliferous whiteflies were then collected for the quantification of TbCSV and TbCSB, and then virus transmission was conducted. Common tobacco plants that were inoculated by whiteflies were sampled for analysis at 18 and 30 dpi. Relative quantities of TbCSV and TbCSB and the TbCSB/TbCSV ratio in whiteflies (**A**–**C**); percentages of symptomatic and TbCSV-positive plants in whitefly-inoculated plants (**D**,**E**); relative quantities of TbCSV and TbCSB and the TbCSB/TbCSV ratio in whitefly-inoculated plants (**F**–**H**). Values are means ± SEM [n = 4 for panels (**A**–**C**); n = 3 for panels (**D**,**E**) and in each replicate 14–15 plants were used; n = 10–17 for panels (**F**–**H**)]. Asterisks indicate significant differences (independent *t*-test, * *p* < 0.05, *** *p* < 0.001) and n.s. indicates no significant difference.

As common tobacco plants at 6 and 18 dpi differed significantly in TbCSB quantity and TbCSB/TbCSV ratio, but not TbCSV quantity, we used these plants as the source of inoculum to examine the effects of TbCSB/TbCSV ratio on whitefly-mediated virus transmission. Whiteflies that fed on plants of the two treatments did not differ in the quantities of TbCSV and TbCSB, but the ratio of TbCSB/TbCSV in whiteflies feeding on plants at 18 dpi was significantly higher than that in whiteflies feeding on plants at 6 dpi (Figure 5A–C). The two groups of whiteflies exhibited significantly different transmission efficiency when indicated by symptom, but not by PCR (Figure 5D,E). In common tobacco plants inoculated by the 2 groups of whiteflies, significant differences in the quantities of TbCSV and TbCSB were found at 30 dpi, but not at 18 dpi (Figure 5F,G). The TbCSB/TbCSV ratio differed significantly between plants inoculated by the 2 groups of whiteflies at 18 dpi, and the difference disappeared at 30 dpi (Figure 5H).

### 3.6. Temporal Dynamics of the Expression of TbCSV AV1 and TbCSB βC1 and the βC1/AV1 Ratio in Common Tobacco Plants

**Figure 6:**
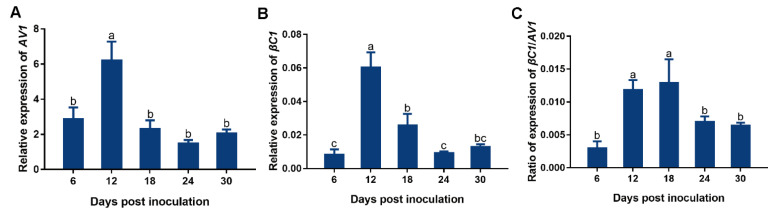
Dynamics of the expression of genes encoded by TbCSV and TbCSB. Common tobacco plants were inoculated with a mixture of equal quantities of agrobacteria containing infectious clones of TbCSV and TbCSB, and then sampled for analysis at designated dpi. Relative expression of *AV1* and *βC1* (**A**,**B**); ratio of expression of *βC1*/*AV1* (**C**). Values are means ± SEM (n = 10–14). Different letters above the columns indicate significant differences (one-way ANOVA, *p* < 0.05).

Relative expression of *AV1* encoded by TbCSV increased significantly from 6 to 12 dpi, decreased significantly from 12 to 18 dpi and then did not show significant changes from 18 to 30 dpi (Figure 6A). Relative expression of *βC1* encoded by TbCSB increased significantly from 6 to 12 dpi, decreased significantly from 12 to 24 dpi and then did not show significant changes from 24 to 30 dpi (Figure 6B). Similarly, the *βC1*/*AV1* ratio in plants increased significantly from 6 to 12 dpi, remained at similar levels from 12 to 18 dpi, decreased significantly from 18 to 24 dpi and then did not show significant change from 24 to 30 dpi (Figure 6C).

### 3.7. Temporal Dynamics of the Quantities of CLCuMuV and CLCuMuB and the CLCuMuB/CLCuMuV Ratio in Plants and Their Response to βC1 Null-Mutation

**Figure 7:**
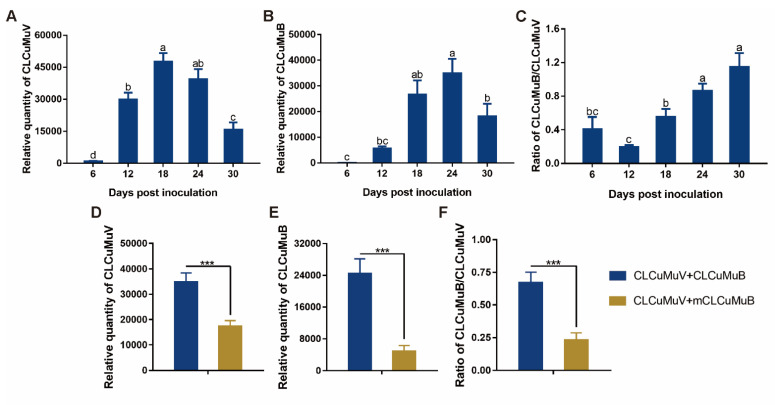
Temporal dynamics of the quantities of CLCuMuV and CLCuMuB and the CLCuMuB/CLCuMuV ratio in plants and their responses to *βC1*-null mutation. *Nicotiana benthamiana* plants were inoculated with a mixture of equal quantities of agrobacteria containing infectious clones of CLCuMuV and CLCuMuB, and then sampled for analysis at designated dpi. Relative quantities of CLCuMuV and CLCuMuB and the CLCuMuB/CLCuMuV ratio in plants (**A**–**C**). mCLCuMuB stands for mutant CLCuMuB. Relative quantities of CLCuMuV and CLCuMuB and the CLCuMuB/CLCuMuV ratio in plants (**D**–**F**). Values are means ± SEM [n = 9–18 for panels (**A**–**C**); n = 14–17 for panels (**D**–**F**)]. In panels (**A**–**C**), different letters above the columns indicate significant differences (one-way ANOVA, *p* < 0.05). In panels (**D**–**F**), asterisks indicate significant differences (independent *t*-test, *** *p* < 0.001).

When *N. benthamiana* plants were inoculated, CLCuMuV quantity increased significantly from 6 to 18 dpi, remained at similar levels from 18 to 24 dpi and then decreased significantly from 24 to 30 dpi (Figure 7A). CLCuMuB quantity increased significantly from 6 to 24 dpi, and then decreased significantly from 24 to 30 dpi (Figure 7B). The CLCuMuB/CLCuMuV ratio did not change significantly from 6 to 12 dpi, increased sequentially from 12 to 24 dpi and did not show significant change from 24 to 30 dpi (Figure 7C). Additionally, *βC1* null-mutation significantly reduced the quantities of CLCuMuV and CLCuMuV and the CLCuMuB/CLCuMuV ratio in plants at 18 dpi (Figure 7D–F).

## 4. Discussion

In this study, we first examined the symptoms induced by TbCSV and TbCSB in common tobacco plants, and found it varies temporally. We then explored the quantitative relationship between TbCSV and TbCSB in natural host common tobacco plants, and the model plant *N. benthamiana*. We showed that in these plants the quantities of TbCSV and TbCSB and the TbCSB/TbCSV ratio varied significantly in the initial 2–3 weeks of infection and thereafter tended to become constant, and this pattern of temporal dynamics was not significantly affected by the TbCSB/TbCSV ratio in agrobacteria inoculum (Figure 2 and Figure 3). *βC1*-null mutation significantly reduced the TbCSB/TbCSV ratio in common tobacco plants in two independent experiments (Figure 4). A higher ratio of TbCSB/TbCSV in inoculated plants promoted virus transmission by whiteflies (Figure 5). The expression of *AV1* encoded by TbCSV and *βC1* encoded by TbCSB varied significantly in the initial 2–3 weeks of infection and thereafter the *βC1*/*AV1* ratio tended to become constant (Figure 6). In addition, we found that in the model plant *N. benthamiana*, the temporal dynamics of the CLCuMuB/CLCuMuV ratio during infection was similar to that of TbCSV and was positively regulated by *βC1* (Figure 7). These results indicate that the betasatellite/begomovirus ratio in virus-infected plants tended to become constant after the initial phase of infection and is modulated by *βC1*, but a higher betasatellite/begomovirus ratio in virally inoculated plants may promote virus transmission by whiteflies.

Agrobacteria-mediated virus inoculation is widely used in the studies of begomoviruses. Using two viruses, we found that after agrobacteria-mediated virus inoculation the betasatellite/begomovirus ratio gradually increased in the initial 2–3 weeks and then remained stable. In another study, when tomato yellow leaf curl virus was inoculated with cotton leaf curl Gezira betasatellite, the ratio of betasatellite/begomovirus gradually increased from 11 to 32 days [25]. These findings suggest that during initial infection, the replication of betasatellites may outpace begomoviruses. Additionally, in later stages, an equilibrium maybe established between the replication of betasatellites and begomoviruses. Similarly, the expression of genes that encoded betasatellites and begomoviruses varied in the initial infection and thereafter an equilibrium of transcription was found. Moreover, this equilibrium does not seem to be affected by the inoculated ratio of betasatellite/begomovirus. The presence of equilibriums indicates the tightly controlled association between begomoviruses and betasatellites. More importantly, our findings highlight that when agroinoculation is used to study begomovirus–betasatellite complexes, the betasatellite/begomovirus ratio in plants should be analyzed and recorded. This parameter may significantly impact the biological characteristics of begomovirus–betasatellite complexes in addition to the nature of begomovirus and betasatellite.

In nature, many plant viruses are multipartite, indicating that they have segmented genome components that are individually encapsidated into separate virus particles [26,27]. For these multipartite viruses, during virus infection, a set-point of relative frequency of viral genomic components (referred to as genome formula) was found [28,29]. Additionally, changes in the relative frequency of viral genomic components were shown to contribute to the regulation of expression of genes encoded by the viral genomic components [26,27,30]. Here, we found that the betasatellite/begomovirus ratio in plants tended to become constant as infection progressed. Moreover, the ratio of the expression of genes encoded by begomovirus and betasatellite became similar as infection progressed, suggesting that the betasatellite/begomovirus ratio in plants may contribute to the regulation of viral mRNA transcription. These findings collectively suggest that monopartite begomoviruses and their betasatellites together behave like multipartite viruses in plants. However, it should be noted that except for a few cotton leaf curl disease-associated viruses, betasatellites are not essentially required in the life history of monopartite begomoviruses [21,31,32,33]. This is in sharp contrast to the relationship between the two genomic components of bipartite begomoviruses [34]. Under this scenario, the evolution of the intimate association between begomoviruses and betasatellites is fascinating and should be subjected to further investigations.

In this study, we found that *βC1*-null mutation significantly reduced the quantities of begomovirus and betasatellite at some of the time points analyzed. This is consistent with the well-established role of the *βC1* gene in the pathogenesis of begomovirus–betasatellite complex [15,19,35]. More interestingly, *βC1*-null mutation significantly decreased the betasatellite/begomovirus ratio in plants. As betasatellites do not encode DNA replicases, their *in planta* replication is believed to be mediated by begomovirus-encoded Rep proteins [5,33]. In this process, the sequences of betasatellites determine the *trans* replication of betasatellites by begomoviral Rep proteins [33,36,37]. For example, a Rep-binding motif (RBM) contributes to the *trans* replication of betasatellites by begomoviral Rep proteins [37]. In our study, we showed that a betasatellite-encoded protein contributes to the regulation of begomovirus–betasatellite association. Instead of abolishing betasatellite replication, *βC1*-null mutation functions to modulate the betasatellite/begomovirus ratio in plants. This may be due to unequal replication and/or degradation of begomoviruses and betasatellites. Further investigations are required to clarify this issue. Nevertheless, our results revealed a novel function of βC1 encoded by betasatellites, namely as a regulator of the quantitative relationship between monopartite begomoviruses and betasatellites in plants.

Taken together, we have found that the ratio between quantities of begomoviruses and betasatellites is temporally dynamic in infected plants, and *βC1* encoded by betasatellites positively regulates the betasatellite/begomovirus ratio in plants. We also showed that a higher betasatellite/begomovirus ratio in inoculated plants promoted virus transmission by whiteflies. Our findings help to understand the nature of the quantitative relationship between begomoviruses and betasatellites, as well as to further decipher the pathogenesis of the begomovirus–betasatellite complex.

## Figures and Tables

**Table 1:** Primers used in this study.

Primer	Sequence (5′-3′)	Application
TbCSV-AV1-RTF	GAAGCGTCCAGCAGATATAA	Quantification of TbCSV and *AV1*
TbCSV-AV1-RTR	GGGACATCAGGACTTCTGTA	
TbCSB-βC1-RTF	AATCACCAGCACTGGCAAAG	Quantification of TbCSb and *βC1*
TbCSB-βC1-RTR	CCGCTTCTTGCATCATCAGG	
Nt-Actin-RTF	GTGTTAGCCACACTGTCCCA	Quantification of *N. tabacum actin*
Nt-Actin-RTR	TCAGTCAAGTCACGACCAGC	
Nb-Actin-RTF	GCGAGTAAACCCGTAAGG	Quantification of *N. benthamiana* actin
Nb-Actin-RTR	GCTCAGGCATAGTTCACC	
TbCSV-A-PCRF	ACAGAAGTCCTGATGTCCCT	PCR detection of TbCSV
TbCSV-A-PCRR	AGAGCACCAGAACCGTCC	
CLCuMuV-AV1-RTF	ACACTTGTGCAGTCCCAGAG	Quantification of CLCuMuV and *AV1*
CLCuMuV-AV1-RTR	CACTTCAACCGTCCATTCAC	
CLCuMuB-βC1-RTF	AGCCGTTGAAGTCGAATGGA	Quantification of CLCuMuB and *βC1*
CLCuMuB-βC1-RTR	GACGAGGAGCAGAACAAACA	
TbCSB-FLF	CCGGAATTCGAAACCACTACGCTACGCAG	Cloning of TbCSB
TbCSB-FLR	CGGGGTACCTACCCTCCCAGGGGTACAC	
TbCSB-TBF	TGTTGTATTTAATTGTCTAATTTGTTCT	Mutagenesis of TbCSB
TbCSB-TBR	TAGACAATTAAATACAACAACAAGAAGGGC	
CLCuMuB-FLF	CGGGGTACCACTACGCTACGCAGCAGCC	Cloning of CLCuMuB
CLCuMuB-FLR	CGGGGTACCTACCCTCCCAGGGGTACAC	
CLCuMuB-TBF	CGGGGTACCTACCCTCCCAGGGGTACAC	Mutagenesis of CLCuMuB
CLCuMuB-TBR	TAGACGAGGAGCAGAACAAACACGCAGG	

## Data Availability

All data supporting this study are included as supplementary information.

## Supplementary Materials

The following supporting information can be downloaded at: https://www.mdpi.com/article/10.3390/v15040954/s1.
